# Exposure to Olfactory Alcohol Cues During Non-rapid Eye Movement Sleep Did Not Decrease Craving in Patients With Alcohol Dependence

**DOI:** 10.3389/fpsyt.2022.837573

**Published:** 2022-04-01

**Authors:** Ran Zhu, Zhaojun Ni, Ran Tao, Jun Cheng, Liangjun Pang, Shun Zhang, Yang Zhang, Yanxue Xue, Yundong Ma, Wei Sun, Lin Lu, Jiahui Deng, Hongqiang Sun

**Affiliations:** ^1^NHC Key Laboratory of Mental Health, Peking University Institute of Mental Health, Peking University Sixth Hospital, Peking University, Beijing, China; ^2^Anhui Mental Health Center, Hefei, China; ^3^Kailuan Mental Health Center, Tangshan, China; ^4^National Institute on Drug Dependence, Peking University, Beijing, China; ^5^Peking-Tsinghua Center for Life Sciences, PKU-IDG/McGovern Institute for Brain Research, Peking University, Beijing, China

**Keywords:** alcohol dependence, sleep, cue exposure therapy, craving, alcohol odor

## Abstract

**Background and Objectives:**

Cue exposure therapy (CET) has been used to reduce alcohol use, but the effect of CET during sleep on alcohol dependence (AD) is unclear. The present study examined the effect of repeated exposure to an olfactory stimulus during non-rapid eye movement (NREM) sleep on cue reactivity and craving in patients with AD.

**Methods:**

Thirty-five patients with AD were enrolled according to the Diagnostic and Statistical Manual of Mental Disorders-IV (DSM-IV). All the subjects were randomly assigned to the experimental or control group. The experimental group was exposed to alcohol odor for 10 min during NREM sleep. The other group (controls) was exposed to water [control stimulus (CtrS)] for 10 min during NREM sleep. Demographic, alcohol-related, and clinical characteristics were collected at baseline. A cue-reactivity test was conducted before and after exposure to evaluate the effect of memory manipulation on acute response to an alcohol stimulus.

**Results:**

There were no significant time × group interactions according to the visual analog scale (VAS) score of craving intensity, skin conductance response (SCR), systolic blood pressure (SBP), and diastolic blood pressure (DBP; all *p* > 0.05). Two-way ANOVA showed significant main effects of time on SCR [*F*_(1,33)_ = 4.453, *p* = 0.043], SBP [*F*_(1,33)_ = 14.532, *p* = 0.001], DBP [*F*_(1,33)_ = 8.327, *p* = 0.007], Craving-VAS [*F*_(1,33)_ = 1.997, *p* = 0.167] in two groups.

**Conclusion:**

Exposure to olfactory alcohol cues during NREM sleep had no significant effect on alcohol craving in subjects with AD during hospitalization.

## Highlights

- There was no significant difference between re-exposure to olfactory alcohol cues or water during NREM sleep in reducing craving and physical-reactivity to alcohol cues in subjects with alcohol dependence (AD).
- A trend toward less physiological reactivity to alcohol cues after sleep over time was observed in all subjects.
- The present study did not yield evidence of the efficacy of exposure cues during sleep in patients with AD.

## Introduction

Alcohol dependence (AD) is an international public health concern (1). Globally, in 2016, 2.6% of people over 15 years of age had AD (2). Although there are many treatments for AD, including pharmacological, behavioral, and psychosocial interventions, many AD patients still experience relapse and poor quality of life because of the chronic character of this disorder, highlighting a critical need to conduct additional research to expand treatment approaches (3).

The crucial question regarding AD treatment is why patients relapse after detoxification despite a strong intention to stay abstinent. Environmental cues are major factors thought to influence behavior and modulate the risk of relapse. Hence, one increasingly dominant view in the addiction literature conceptualizes the disease as one of aberrant learning and memory (4–6). Maladaptive motivational memory (MMM) associations link conditioned stimuli (CS; e.g., people, places, or bottles) with the intoxicating and rewarding effects of unconditional stimuli (US; e.g., alcohol) (7, 8). US and CSs contribute significantly to craving and subsequent alcohol consumption behaviors (9–11). Once formed, these MMMs are difficult to resolve because they are highly robust and resistant to destabilization.

Based on the classical conditioning theory, cue exposure therapy (CET) was designed to induce memory extinction through repeated exposure to CS without alcohol consumption to decrease conditioned responses (12). Several studies have reported positive effects of CET on reduced alcohol consumption (13), reduced craving (13), prolonged abstinence, and decreased cue reactivity (14). However, in a recent meta-analysis on CET targeting alcohol use disorder (AUD), CET showed no effect on drinking-related outcomes, and CET had a moderate effect on relapse (15). There are several possible reasons for such discrepant results. Notably, memory extinction is a largely passive procedure involving exposure to environmental cues, worsening negative responses by making the subjects recall painful experiences.

A single night of aversive olfactory conditioning during non-rapid eye movement sleep (NREM) sleep stage 2 (N2) reduced cigarette consumption in smokers (16). This suggests that sleep might interfere with substance-related MMMs. In seminal works, target memory reactivation (TMR) during sleep, similar to CET during wakefulness, reduced fear responses through memory extinction and augmented the efficiency of CET (17, 18). These findings support the hypothesis that new learning during N2 sleep or slow-wave sleep (SWS) can modify memory and alter later behaviors. The TMR paradigm poses a challenge to traditional CET, as sleep induces an anesthesia-like state during which a patient’s bad memory can be eliminated to optimize cognitive and behavioral therapy.

In a recent meta-analysis, the researchers found that TMR was only significant during the two NREM stages: N2 and SWS (19). Research about odor re-exposure during sleep found increased left-lateralized frontal slow spindle (11.0–13.0 Hz) and right-lateralized parietal fast spindle (13.0–15.0 Hz) activity, suggesting the possibility of a successful re-presentation of therapy-related memories during sleep (20). Specifically, during NREM sleep featuring cortical slow oscillations (SO) and thalamocortical spindles, covert memory reactivation can transform newly acquired, hippocampus-dependent learning such that neocortical representations become more stable and resistant to disruption (19). Furthermore, recent studies emphasized the importance of stage 2, spindles and the SO-spindle coupling in procedural memory consolidation (21). NREM sleep might be an excellent time window to enhance the effect of TMR. Notably, the patients with alcohol dependence have reported decreased SWS and increased latency of N2 sleep. A study indicated that N2 sleep during withdrawal accounts for 30% of total sleep time in alcohol-dependent patients, while SWS accounts for only 6.7% of total sleep time (22). Unlike healthy individuals, the reduction in sleep time might result in SWS sleep exposure alone not being able to provide stimulus exposure of sufficient duration.

Therefore, to elucidate whether repeated exposure to cues during sleep can be applied in a real clinical setting to reduce alcohol craving or consumption, we conducted a controlled study to determine the effects of re-exposure to an olfactory alcohol cue during NREM sleep in subjects with AD. Therefore, we chose to deliver odor cues at the N2 stabilization session and to continue the presentation when shifting to the N3 phase to ensure 20 min of cue exposure for extinction (23), in line with previous studies (20, 24). We hypothesized that repeated exposure to olfactory alcohol cues during NREM sleep would decrease alcohol craving and related physiological indexes in AD patients.

## Materials and Methods

### Participants

All participants were recruited from psychiatric hospitals (Peking University Sixth Hospital, Beijing, and Anhui Mental Health Center, Hefei) in China. The inclusion criteria were as follows: (1) male; (2) 18–60 years old; (3) AD diagnosed according to the Diagnostic and Statistical Manual of Mental Disorders-Fourth Edition (DSM-IV) (25) and assessed by the Mini-International Neuropsychiatric Interview (M.I.N.I.) (26); (4) within 14–45 days after hospital admission and not in acute clinical withdrawal from alcohol as verified by a Clinical Institute Withdrawal Assessments for Alcohol, revised version (CIWA-Ar) (27) score of <7; and (5) able to fall asleep at a regular hour according to the hospital sleep requirements. The exclusion criteria were as follows: (1) diagnosed with other Axis I psychiatric disorders and any previous or current substance dependence other than nicotine use according to the DSM-IV; (2) significant medical conditions (e.g., cardiovascular or cerebrovascular disease); (3) dysosmia, recent (<4 weeks) nasal infection, or any self-reported difficulty with smell; or (4) use of any pharmacological treatment to reduce alcohol craving, such as acamprosate, aripiprazole, quetiapine, baclofen, or nalmefene. Written consent was obtained from all subjects before obtaining any study measurements. The study was approved by the Ethics Committee of Peking University Sixth Hospital, Beijing, China.

**FIGURE 1:**
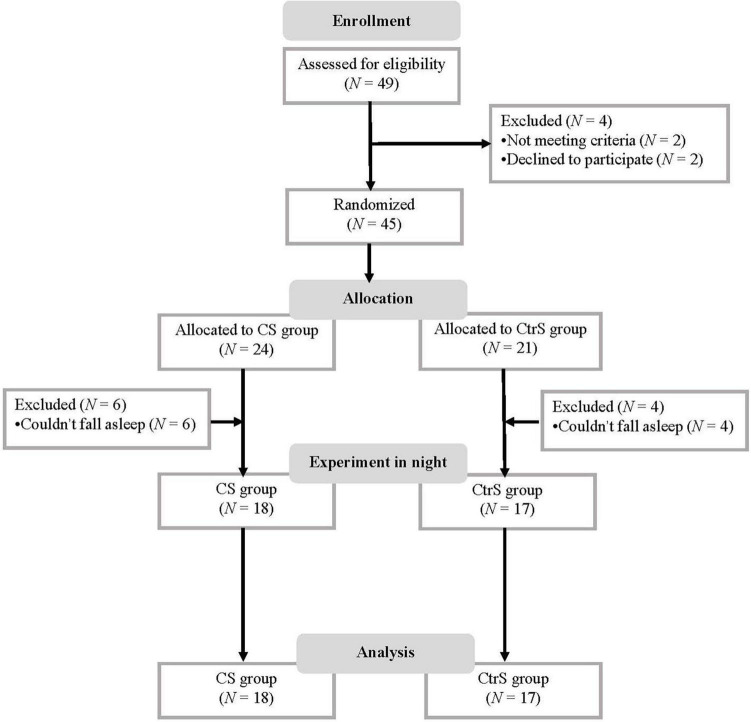
Flow diagram. CS group, patients was re-exposed to olfactory alcohol cues; CtrS group, patients was re-exposed to water as an olfactory control.

A total of 49 patients with AD were screened between September 2017 and December 2018. Forty-five participants were randomly assigned to two groups. The final sample consisted of 35 patients in two groups: The sleep with an olfactory alcohol stimulus (CS) group (*n* = 18) and the sleep with a water stimulus (CtrS) group (*n* = 17). The flow diagram of the enrollment process is illustrated in Figure 1.

### Procedures

**FIGURE 2:**
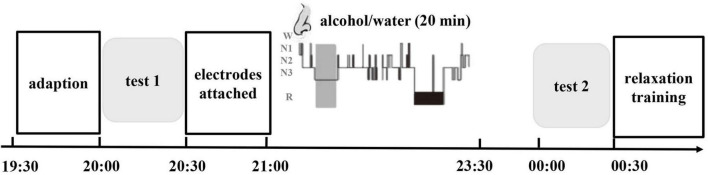
Timeline of the experimental methods. The experimental protocol consisted of three phases: the baseline cue-reactivity test (test 1), extinction during sleep and recall test (test 2). A 2.5-h interval of nocturnal sleep between the initial test and later retesting was allowed. During the first stable NREM sleep period, one group was re-exposed to olfactory alcohol cues (CS group), and the other group was re-exposed to water as an olfactory control (CtrS group). The light gray areas represent the cue reactivity test, and the dark gray area represents the first period of NREM sleep when the odor stimulus was delivered.

The study consisted of two main phases: (1) a baseline evaluation conducted by a professional psychiatrist during the daytime and (2) exposure to cues during sleep. Before and after exposure to the intervention, we assessed the subjective and physical responses to alcohol-related stimuli during a test with olfactory alcohol cues (test 1 and test 2). The patients in the two groups were invited to participate in baseline evaluations and cue testing (test 1). Then, all subjects were asked to participate in test 2 after sleep intervention. During the period of hospitalization, the subjects had no access to alcohol. In addition, all the subjects underwent detoxification and rehabilitation in the inpatient unit. The experimental procedures are illustrated in Figure 2.

#### Baseline Measurements

The subjects participated in an interview to obtain information about their sociodemographic, alcohol consumption-related, and clinical characteristics, including age, sex, marital status, education level, body mass index (BMI), favorite alcoholic beverage in the last year, duration of alcohol dependence, days of abstinence before baseline measurement, and daily alcohol intake (standard drinks). The Self-rating Anxiety Scale (SAS) (28) and Self-rating Depression Scale (SDS) (29) were used to assess the level of intensity of anxiety and depression during withdrawal, respectively. The Alcohol Urge Questionnaire (AUQ) (30) was used to evaluate the level of intensity of alcohol craving during the abstinent period. Sleep quality was assessed by the Pittsburgh sleep quality index (PSQI) (31). The Montreal Cognitive Assessment (MoCA) (32) was used to evaluate cognitive function. The Fagerstrom Test for Nicotine Dependence (FTND) (33) were used to assess dependence on nicotine.

#### Behavioral Paradigm

In each experiment, after a break at 19:30, participants were brought to the operating room, which was maintained at 25°C. Initially, subjects were instructed to relax and adapt for 30 min. All subjects were assessed for cue reactivity (test 1) between 20:00 and 20:30 before sleep onset, and then PSG electrodes and a small nasal mask for odor delivery were applied. Because odorant stimuli are sensory cues that can be administered unobtrusively during sleep and favor subsequent extinction of maladaptive memories, we selected alcohol odor as the cue during NREM sleep (34).

To avoid the possibility of confounding effect of rapid eye movement (REM) on memory (35), we used a similar “night-half paradigm,” which was deemed NREM-rich sleep (18). The subjects were allowed to sleep from 21:00 to 23:30, as well as consistent with subjects’ sleep schedule during treatment. We presented the odor as soon as the online PSG recordings indicated stable N2 sleep with visible K-complexes for 2 epochs. Immediately before test 2, participants recorded their subjective alertness levels using the Stanford Sleepiness Scale (SSS) (36). Subjects were asked whether they had smelled the odor (“yes”, “no,” or “don’t know”) (37). Then, they underwent again the cue recall test, which was the same as test 1.

#### Cue Reactivity Assessment

All subjects were requested to evaluate the intensity of their craving on a 10-point Visual Analog Scale (VAS) ranging from 0 (not at all) to 10 (extremely high). Thereafter, systolic blood pressure (SBP) and diastolic blood pressure (DBP) were collected as baseline responses. An electronic blood pressure monitor (OMRON, HBP-1300) was used to assess blood pressure. Next, the subjects were fitted with two Ag-AgCl electrodes attached to the middle phalanges of the second and third fingers of the non-dominant hand. A multichannel biofeedback device (Thought Technology Ltd., Montreal, QC, Canada, VBFB3000) was used to monitor the skin conductance response (SCR). To avoid movement artifacts, each participant was instructed to keep the hand connected to the electrodes still and relaxed; the hand rested on the arm of the testing chair throughout the whole test. A typical alcohol bottle was placed on the table directly in front of the participants; the subjects were instructed to handle and smell the alcohol bottle for 60 s without consuming it (14). The experimenters asked the participant to estimate the intensity of craving on the VAS again. The physiological measures collected included SCR and blood pressure (BP). The amplitude of the SCR to the alcohol cue was determined by calculating the peak-to-base difference within a 60 s window following the onset of odor delivery (i.e., max SCL-min SCL during exposure to alcohol cue). The baseline value was the mean SCR during the 2 s before stimulus exposure (20, 24). Successful cue-reactivity test was evidenced by increased stimulus-evoked SCR for alcohol odor cue in comparison to the baseline (17). Immediately after the test, BP was once again collected. The primary outcome measure was the change in cardiovascular response, SCR, and VAS score. All subjects underwent two cue-reactivity tests before (test 1) and after (test 2) exposure.

#### Polysomnographic Recordings

We used standard polysomnography (PSG) to record sleep. Electroencephalography (EEG) was performed using six scalp electrodes (C3, C4, F3, F4, O1, and O2 according to the international 10–20 system) referenced to a contralateral mastoid electrode. EEG signals were filtered between 0.3 and 35 Hz and sampled at 256 Hz. 30 s epochs were used for manual analysis. In addition to the online identification of sleep stages, EEG was scored offline according to the criteria of the American Academy of Sleep Medicine (38). Sleep stages scored include total sleep time, sleep latency, stage 1 sleep, stage 2 sleep, SWS, and REM sleep.

#### Olfactory Stimulus Delivery

The odorant stimulation was delivered by a respirator via a nasal mark. The alcohol odorant was diluted in purified water at a concentration of 1:50. Alcohol odors or odorless stimuli (purified water) were presented using a 30 s on/30 s off schedule for 20 min. We conducted the experiment with no instructions or information provided to the subjects regarding odor presentation during sleep. The extinction-associated odor stimulus (alcohol odor) or odorless stimulus (water) was presented when we identified 2 consecutive N2 epochs. The odor stimulus was terminated when 20 trials (30 s on/30 s off schedule) were completed. If microarousal occurred, the stimulus that evoked the microarousal was not counted in the total number of stimuli. Presentations continued when participants transitioned into N3 but were stopped immediately upon the slightest sign of arousal, awakening, or REM sleep (24, 39). After all the subjects awakened, we asked whether they perceived the odor. Approximately 30 min after the subjects woke up or had remained awake for the same time interval, the level of sleepiness was evaluated with the SSS.

### Analysis

The demographic and clinical traits, baseline craving levels, and physiological characteristics of the two groups were assessed using independent *t*-tests or chi-square tests. For measures obtained during the testing sessions, all values of the cue-reactivity tests, including the VAS score, SCR, and cardiovascular response, are presented as score changes and were calculated by subtracting the precue reactivity value from the post cue reactivity value during test 1 and test 2. These results were analyzed using repeated-measures ANOVAs, with group (CS group and CtrS group) as the between-subjects factor and time (test 1 and test 2) as the within-subjects factor. We performed *post hoc* analyses of significant effects in the ANOVAs using the Bonferroni method. Two-sided of *p-*values < 0.05 were considered statistically significant. Statistical analyses were carried out using SPSS version 24.0 (IBM, Armonk, NY, United States) software for Windows.

Power estimates for the current study were limited, as no studies to date have examined within-group effects of exposure to olfactory alcohol cues during sleep on reducing cue-reactivity among patients with AD. Consequently, the sample size was calculated based on a.05 alpha level, revealing that 80% power (1-β) would allow detection of a significant within-group effect of exposure observations via F-testing to assess a medium to large effect size (0.15 ≤ effect size *f*^2^ ≤ 0.35). This yielded a total required sample size of 20–90 subjects (G*power 3.1.9.2). Given these power approximations and limited resources, we estimated that our study sample of 35 individuals with repeated exposure to alcohol cues during sleep was appropriately powered to detect the hypothesized effect.

## Results

### Demographic Characteristics

The participants comprised 35 individuals with AD, with a mean age of 38 years (SD = 7.9). There were no significant differences in demographic or alcohol-related characteristics between the two groups at baseline (all *p* > 0.05; Table 1).

**TABLE 1:** Demographic and clinical characteristics of the subjects in the CS and CtrS groups.

Variables	CS group (*n* = 18)	CtrS group (*n* = 17)	*p*-value
Age (years)	38.00 ± 5.83	38.69 ± 8.63	0.892
Education (years)	10.67 ± 3.02	12.44 ± 3.44	0.631
**Marital Status**			0.394
Married	13 (72.2%)	13 (76.5%)	
Single	0 (0%)	1 (5.9%)	
Divorced or widowed	5 (27.8%)	3 (17.6%)	
BMI (kg/m^2^)	23.24 ± 3.40	22.91 ± 2.81	0.689
Abstinence before baseline measurement (days)	23.92 ± 8.10	21.38 ± 8.21	0.237
Duration of AD (years)	7.25 (4.25–8.75)	4.00 (3.00–5.75)	0.148
Daily alcohol intake (standard drinks)	20.90 ± 7.36	17.35 ± 6.64	0.436
Number of previous inpatient withdrawal treatments	2.00 (1.00–6.00)	2.00 (1.00–3.00)	0.833
MoCA score	26.08 ± 1.19	26.69 ± 1.89	0.551
CIWA-Ar score	1.00 (1.00–4.00)	1.50 (1.00–5.75)	0.393
PSQI score	7.25 ± 4.07	7.97 ± 3.75	0.899
FTND score	5.50 (3.25–7.00)	3.50 (1.25–5.75)	0.140
SAS score	33.33 ± 6.53	31.75 ± 6.46	0.275
SDS score	38.25 ± 11.06	36.00 ± 6.20	0.600
SSS score	3.00 (2.00–3.00)	3.00 (2.25–3.00)	0.091
**Antipsychotic**			
Yes	12 (66.7%)	12 (70.6%)	0.803
No	6 (33.3%)	5 (29.4%)	
**Antidepressant**			
Yes	14 (77.8%)	12 (70.6%)	0.627
No	4 (22.2%)	5 (29.4%)	
**Sedative-hypnotic**			
Yes	3 (16.7%)	2 (11.8%)	0.679
No	15 (83.3%)	15 (88.2%)	

*The data are expressed as the mean ± standard deviation or median and interquartile range or frequencies. AD, alcohol dependence; BMI, body-mass index; MoCA, Montreal Cognitive Assessment; CIWA-Ar, Clinical Institute Withdrawal Assessment of Alcohol Scale, Revised; PSQI, Pittsburgh Sleep Quality Index; FTND, Fagerstrom Test for Nicotine Dependence; SAS, Self-rating Anxiety Scale; SDS, Self-rating Depression Scale; SSS, Stanford Sleepiness Scale.*

### Polysomnographic Results

Table 2 showed that re-exposure to alcohol cues or control cues during NREM sleep did not affect sleep profiles (total sleep time, *p* = 0.932; stage 1 sleep, *p* = 0.143; stage 2 sleep, *p* = 0.234; SWS, *p* = 0.378; REM sleep, *p* = 0.514; sleep efficiency, *p* = 0.701).

**TABLE 2:** Sleep parameters in the CS and CtrS groups.

Variables	CS group (*n* = 18)	CtrS group (*n* = 17)	*p*-value
Total sleep time (min)	34.25 (24.75–50.63)	31.75 (24.63–38.13)	0.932
Sleep latency (min)	14.25 (3.13–44.63)	34.00 (19.63–48.25)	0.242
Stage 1 sleep (min)	1.25 (0.63–2.38)	0.50 (0–1.38)	0.143
Stage 2 sleep (min)	21.00 ± 8.25	16.88 ± 8.27	0.234
SWS (min)	5.25 (0.13–20.88)	12.00 (1.13–23.00)	0.378
REM sleep (min)	0 (0–1.50)	0	0.514
Sleep efficiency	0.42 ± 0.18	0.39 ± 0.13	0.701

*The data are expressed as the mean ± standard deviation or median and interquartile range. SWS, slow-wave sleep; REM, rapid eye movement.*

### No Effect of Exposure on Subjective Cue Reactivity

**FIGURE 3:**
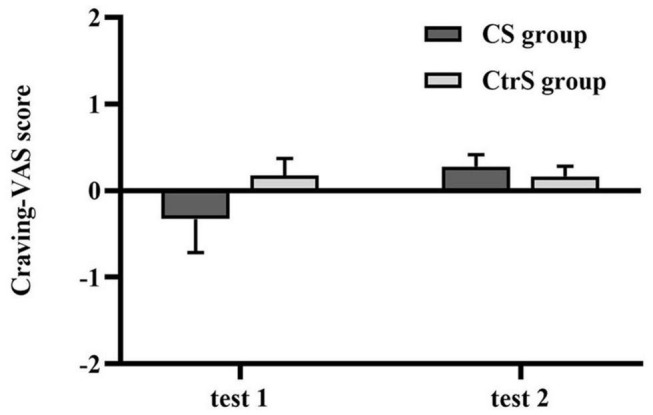
Cue-induced alcohol craving (mean ± SEM) at baseline (test 1) and retest (test 2) for the CS group and CtrS group. These ratings were obtained from the VAS measures and were presented as change scores in craving. None of the differences were statistically significant.

There was no significant difference in the craving for alcohol by VAS between the two groups at baseline (*p* = 0.972; Table 3). Despite the upward trend in craving for each group, the analysis showed no significant main effects of time [*F*_(1,33)_ = 1.997, *p* = 0.167] or a group × time interaction [*F*_(1,33)_ = 2.158, *p* = 0.151]. In our study, the subjects returned to baselines levels before being tested again at the second time point. The craving scores of the two cue-reactivity tests are shown in Figure 3.

**TABLE 3:** Baseline subjective and physiological indicators in the CS and CtrS groups.

Variables	CS group (*n* = 18)	CtrS group (*n* = 17)	*Z*	*p*-value
SCR mean	1.12 (0.88–1.46)	0.74 (0.47–1.43)	−1.520	0.128
SBP	137.00 (125.00–155.50)	133.00 (119.15–137.00)	−1.535	0.125
DBP	82.50 (75.25–94.25)	82.00 (67.30–90.15)	−1.023	0.306
Craving by VAS	0.30 (0–4.00)	0.20 (0–2.70)	−0.035	0.972

*The data are expressed as median and interquartile range. SCR, skin conductance response, the baseline value was the mean SCR during the 2 s before stimulus exposure; SBP, systolic blood pressure; DBP, diastolic blood pressure; VAS, analog scale.*

### Skin Conductance Responses Decreased in Both the Conditioned Stimuli and Control Stimuli Group

**FIGURE 4:**
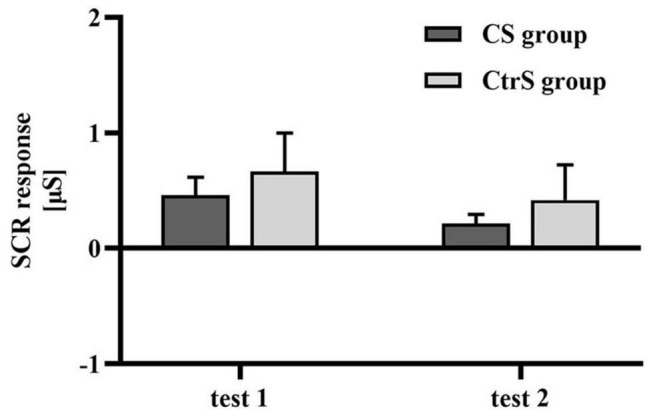
Cue-induced SCR changes (mean ± SEM) within two cue reactivity tests for the CS group and CtrS group. SCR in both groups were significantly lower than those before intervention.

The baseline value was the mean SCR during the 2 s before stimulus exposure, and there was no significant difference in the mean SCR between the two groups at baseline (*p* = 0.128; Table 3). Regarding physiological responses to alcohol cues, repeated-measures ANOVAs, with group (CS group and CtrS group) as the between-subjects factor and time (test 1 and test 2) as the within-subjects factor, showed a significant main effect of time on SCR [*F*_(1,33)_ = 4.453, *p* = 0.043], and no main effect of group [*F*_(1,33)_ = 0.425, *p* = 519; Figure 4].

### Changes in Cardiovascular Measures Over Time

**FIGURE 5:**
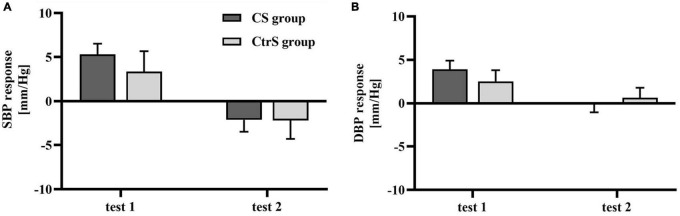
Cue-induced cardiovascular changes within two cue reactivity tests for the CS group and CtrS group. **(A)** SBP changes. SBP responses in both groups were significantly lower than those before intervention. **(B)** DBP changes. DBP responses in both groups were significantly lower than those before intervention.

There were no significant differences in the SBP and DBP between the two groups at baseline (*p* = 0.125 and *p* = 0.306, respectively; Table 3). However, BP was different between the two cue-reactivity tests. A main effect of time was observed for SBP [*F*_(1,33)_ = 14.532, *p* = 0.001], with lower SBP in the post-exposure cue reactivity test. Similarly, a main effect of time was observed for DBP [*F*_(1,33)_ = 8.327, *p* = 0.007], with lower DBP in the post-exposure cue reactivity test. However, the group × time interaction had no effect on either SBP or DBP [*F*_(1,33)_ = 0.308, *p* = 0.980; *F*_(1,33)_ = 1.069, *p* = 0.309; Figures 5A,B].

## Discussion

To our knowledge, this was the first study to explore the effect of re-exposure to olfactory alcohol cues during NREM sleep on alcohol craving and physiological responses in subjects with AD. We found that after sleep, repeated exposure to an alcohol stimulus during NREM sleep was ineffective in reducing subjective craving or physical responses, including changes in the SCR and blood pressure, in the CS group, contrary to our *a priori* hypothesis.

Unexpectedly, studies on memory manipulation during sleep have reported contrasting results. Because of the paucity of literature on TMR in drug users, we referred to related studies on both fear and drug memories, focusing on the effect of TMR similarly to CET. One study promoted fear extinction, in which participants underwent re-exposure to the odorant stimulus during SWS; this was more efficient than repeated exposure to cues during wakefulness (17). Another study reported re-exposure to cues during SWS reinstated fear responses in humans (24), and some animal studies also found enhanced fear responses (40, 41). Given the replication crisis in the fear memory field, recent papers have cautioned about the lack of standardization of procedures, and it is possible that the significant findings within the field of psychology are exaggerated (42). In previous human fear memory studies, fear conditioning training was conducted in healthy subjects (23), whereas our study took advantage of pre-existing addiction memory. We recruited alcohol-dependent patients in an attempt to explore the effects of TMR on maladaptive memories. However, unlike a learned fear memory, maladaptive drug-related memories are learned over hundreds of thousands of trials in multiple contexts and therefore likely to be highly resistant to destabilization and extinction (43). Moreover, memory extinction just creates a novel association between only special CS and US to compete with maladaptive alcohol memories. As a largely passive procedure, extinction training lacks reinforcement. Counterconditioning, which re-pairs reward cues (e.g., pictures of beer) with negative outcomes (e.g., disgust-inducing bitter liquids and odors), may provide a more powerful corrective learning experience than extinction (44). In seminal work, researchers found that olfactory aversive conditioning during N2 sleep resulted in a greater reduction in smoking behavior that lasted for a longer time than the reduction following similar conditioning in REM sleep (16). A recent study highlighted greater long-term reductions in alcohol consumption when counterconditioning was conducted following retrieval (4).

In addition, it is worth considering whether some aspect of the design might reduce the efficacy of TMR. The length of exposure is likely to influence the efficacy of extinction training. During this NREM sleep period, we delivered odor cues to participants for 20 min, resulting in each cue being presented multiple times (20 presentations). Previous research using tone cues suggested that the number of sound replays is not important for benefits of TMR to emerge (36). Furthermore, TMR benefits have been observed following the presentation of each cue only once during the sleep period (45). Some researchers have also pointed out that it is possible that amassed presentation of the cues within 3–40 min leads to extinction, whereas presenting single cues over a longer time period of 2–4 h strengthens the underlying memory trace (23).

Another possibility is the lack of sensitivity of acute laboratory assessment of the reinforcing effects of alcohol since self-reported craving might not truly reflect the urge to drink. Alcohol-related olfactory cues may be powerful appetitive cues, eliciting a craving response (34). In test 1, alcohol cues elicited strong physical reactivity but not a subjective craving increase in both groups (Supplementary Table 1). Participants who were re-exposed to alcohol cues or water during sleep all showed a trend toward a stronger craving for alcohol in test 2, but the difference was not significant in either group. It has been difficult to assess craving in clinical settings, and there have been inconsistent conclusions regarding the association of craving with relapse (46). We used the VAS to assess the desire for alcohol because of its ease of use in facilitating multiple evaluations of craving. VASs are easy-to-use measurement tools, but they are not as accurate as multi-item questionnaires. In comparison to multi-item questionnaires, subjects report less nuanced ratings on a unidimensional scale. Furthermore, given that craving on VAS was rated during hospitalization, when subjects were undergoing psychosocial and pharmacological treatment that may have interfered with the assessment of craving, lower VAS scores seem possible. However, to exclude the limitations of evaluating subjective craving via the VAS, we assessed the SCR and cardiovascular measures. Throughout NREM sleep, the SCR and blood pressure responses to cues both decreased over time in each group. The SCR and blood pressure showed strong generic habituation over repeated stimulus presentation. It is plausible that we found no effect of memory manipulation due to the lack of accurate measurement indexes.

Importantly, some researchers have argued that the role of individual differences has been neglected in behavior studies (10). In the context of individual differences, whether each subject can perceive cues, beyond strengthening existing associated memory, and forming new memory (i.e., memory extinction) require to expand studies. On a neural basis, hippocampal reactivation is suggested as the underlying mechanism behind consolidation during sleep. Nevertheless, little is known about the precise time course and boundary conditions of fading of hippocampal representations (47). Accordingly, the lag between learning and the posttest is also a factor that may explain the inconsistent results. To date, parametric studies on memory reactivation are lacking, and it is unclear which parameters are optimal.

There were several limitations to our study. First, the sample was relatively small, and patients were enrolled during hospitalization. Large sample size and randomized controlled studies to investigate the effect of paradigm during sleep on AD are needed in the future. The study lacked the power to detect potential effects of re-exposure to alcohol cues during sleep. Second, in our paradigm, we did not probe the role of other sleep stages in memory (47). REM sleep is known to contribute to memory consolidation (48). In both rodent and human studies, it has been reported that REM sleep deprivation was associated with fear memory recall (49–51). Even so, a recent meta-analysis of TMR provided substantial support for the notion that TMR was not effective during REM sleep or wakefulness (19). Also, we did not explore the respective effects of N2 and SWS sleep periods on MMMs. Stimulation with learning-associated cues, mainly during SWS, has been shown to enhance declarative as well as procedural and emotional types of memory (17, 52, 53). Although the Meta-analysis reported TMR during SWS benefits on memory (19), recent work also suggested no effect of TMR on the recognition of emotionally negative or neutral memories (54). Due to the importance of stage 2, spindles and the SO-spindle coupling in memory consolidation (21), the relationship and different roles of NREM and SWS in the TMR are still unclear and need to be explored in future studies. Cue exposure might activate the addiction memory trace, leading to reduced cue response test result in the same context when tested immediately after sleep. Moreover, sleep itself might restabilize the trace during the short term. Additionally, we did not perform a similar procedure during the daytime to investigate the effect of CS exposure during wakefulness, which would determine whether the paradigm we used could prevent craving in patients with AD. Third, alcohol odor delivery was hand controlled, but the use of a respirator induced a delay in the experimenter’s reaction time. Although many studies on odor cueing used a computer-controlled olfactory meter to deliver odor, it was challenging to persuade clinicians to use the machine in real clinical settings. These limitations could be overcome in the future. Finally, our sample comprised only men. Therefore, the generalization of our conclusions to female patients with AD is limited. As previous studies have shown that cue-induced alcohol craving and physiological cue reactivity are associated with alcohol relapse risk and that well-tolerated re-exposure to cues during sleep could facilitate extinction memory, the hypothesis in the current study requires further testing to evaluate effects on alcohol craving and improve treatment outcomes.

## Conclusion

To our knowledge, the present study is the first to examine the effect of CS re-exposure during NREM sleep on addiction memory in individuals with AD. We observed no impact of re-exposure to alcohol cues during NREM sleep on cue-induced craving and physiological response. The replication crisis in psychology highlights the need for further examination of factors that may moderate memory extinction manipulation effects and the lack of standardized procedures.

## Data Availability Statement

The raw data supporting the conclusions of this article will be made available by the authors, without undue reservation.

## Ethics Statement

The studies involving human participants were reviewed and approved by the Ethics Committee of Peking University Sixth Hospital, Beijing, China. The patients/participants provided their written informed consent to participate in this study.

## Author Contributions

RZ, ZN, RT, YX, YM, WS, JD, and HS contributed to the study conception and design. RZ, SZ, JC, and LP evaluated all the patients, performed the data collection, and conducted the study. YZ and WS conducted the sleep data scoring. RZ and JD performed the data analyses. RZ drafted the manuscript. LL and HS critically revised the important intellectual content in the manuscript. JD and HS were in charge of supervision. All authors read and approved the final manuscript.

## Conflict of Interest

The authors declare that the research was conducted in the absence of any commercial or financial relationships that could be construed as a potential conflict of interest.

## Publisher’s Note

All claims expressed in this article are solely those of the authors and do not necessarily represent those of their affiliated organizations, or those of the publisher, the editors and the reviewers. Any product that may be evaluated in this article, or claim that may be made by its manufacturer, is not guaranteed or endorsed by the publisher.
