# Supplementary material for: Exposure to Olfactory Alcohol Cues During Non-rapid Eye Movement Sleep Did Not Decrease Craving in Patients With Alcohol Dependence
Source: Front Psychiatry. 2022 Apr 1;13:837573. doi: 10.3389/fpsyt.2022.837573 (PMC9010533; doi:10.3389/fpsyt.2022.837573)
Supplement: Supplementary file 1 [file Table_1.pdf]

Supplementary information for:

Exposure to olfactory alcohol cues during NREM sleep did not decrease craving in patients with alcohol dependence

Ran Zhu[1], Zhaojun Ni[1], Ran Tao[1], Jun Cheng[2], Liangjun Pang[2], Shun Zhang[3], Yang Zhang[1], Yanxue Xue[4], Yundong Ma[1], Wei Sun[1], Lin Lu[1,4,5], Jiahui Deng[1,*], Hongqiang Sun[1,*]

1 Peking University Sixth Hospital, Peking University Institute of Mental Health, NHC Key Laboratory of Mental Health (Peking University), National Clinical Research Center for Mental Disorders (Peking University Sixth Hospital), 51 Huayuan Bei Road, Haidian District, Beijing, 100191, China
2 Anhui Mental Health Center, 316 Huangshan Road, Shushan District, Hefei, 230001, China
3 Kailuan Mental Health Center, 7 Huayuan Street, Lunan District, Tangshan, 063001, China
4 National Institute on Drug Dependence, Peking University, 38 Xueyuan Road, Haidian District, Beijing, 100191, China
5 Peking-Tsinghua Center for Life Sciences and PKU-IDG/McGovern Institute for Brain Research, Peking University, Beijing, 100191, China

[*]Corresponding authors:

Prof. Hongqiang Sun, M.D., Ph.D, Peking University Sixth Hospital/Peking University Institute of Mental Health, NHC Key Laboratory of Mental Health (Peking University), National Clinical Research Center for Mental Disorders (Peking University Sixth Hospital), 51 Huayuan Bei Road, Haidian District, Beijing 100191, China.
Tel: +86-10-8280 5960
Fax: +86-10-6202 6310
E-mail: sunhq@bjmu.edu.cn
Dr. Jiahui Deng, Ph.D, Peking University Sixth Hospital/Peking University Institute of Mental Health, NHC Key Laboratory of Mental Health (Peking University), National Clinical Research Center for Mental Disorders (Peking University Sixth Hospital), 51 Huayuan Bei Road, Haidian District, Beijing 100191, China.
Tel: +86-10-6272 3707
Fax: +86-10-6203 2624
E-mail: jiahuideng2012@bjmu.edu.cn

Supplementary Table 1. **Cue-induced craving and physiological reactions for alcohol odor cue during test 1.**

Variables	Pre-test 1 (n = 35)	Post-test 1 (n = 35)	Z	p value
SCR	0.02 (0.01–0.04)	0.15 (0.04–0.58)	-4.470	< 0.001
SBP	134.70 (122.00–146.00)	137.00 (121.00–152.25)	-2.975	0.003
DBP	82.00 (75.00-92.00)	86.00 (75.00–96.00)	-3.265	0.001
Craving by VAS	0.20 (0–2.70)	0 (0–2.85)	-0.739	0.460

The data are expressed as median and interquartile range.

Abbreviations: SCR, skin conductance response; SBP, systolic blood pressure; DBP, diastolic blood pressure; VAS, visual analog scale.
